# The Unique Bipolar Clavicle Dislocation: A Novel Reconstruction Technique and Case Report

**DOI:** 10.1155/2020/8888818

**Published:** 2020-08-10

**Authors:** Ameesh Dev, Gautham Prabhakar, Anil Dutta, Khang Dang

**Affiliations:** Department of Orthopaedic Surgery, UT Health San Antonio, San Antonio, TX, USA

## Abstract

A bipolar clavicle separation is defined as a simultaneous dislocation of the ipsilateral sternoclavicular joint (SCJ) and acromioclavicular joint (ACJ). This rare injury pattern is usually the result of a high-energy mechanism, such as a motor vehicle collision or fall from height. While there are several treatment options such as screw fixation, sutures, or plate fixations, there is no single standard approach for this infrequent injury. We describe a unique case of bipolar clavicle dislocation, specifically an anteriorly displaced SCJ and posteriorly displaced ACJ, treated with a novel surgical technique—a TightRope technique (Arthex®) and semitendinosus allograft.

## 1. Introduction

Usually resulting from high-energy trauma, bipolar clavicle dislocations, also known as “floating” clavicles, occur when the clavicle simultaneously dislocates from the acromioclavicular joint (ACJ) and the sternoclavicular joint (SCJ) [1–3]. The mechanism of injury is the result of a force to the lateral affected shoulder or medial compression of both shoulders [3]. Understanding the injury pattern is critical for the appropriate treatment to maximize functional and clinical outcomes. Treatment options for ACJ and SCJ dislocations include observation, K-wires [3, 4], cerclage wires [5], compression screws [6], polyester mesh [7], hook plates [8], and FiberWire [2]. However, the ideal treatment choice is still controversial, and a gold standard does not currently exist. To our knowledge, our case is the first documented case report of a bipolar clavicle dislocation-fracture, in which the ACJ was successfully treated with the Arthex® TightRope and the SCJ with a semitendinosus allograft.

## 2. Case Presentation

**Figure 1:**
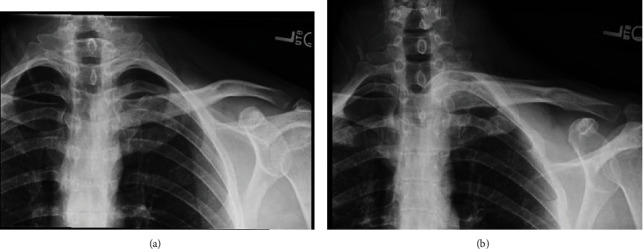
(a, b) Plain radiographs demonstrating anterior dislocation of the sternoclavicular joint as well as a posterior acromioclavicular dislocation.

**Figure 2:**
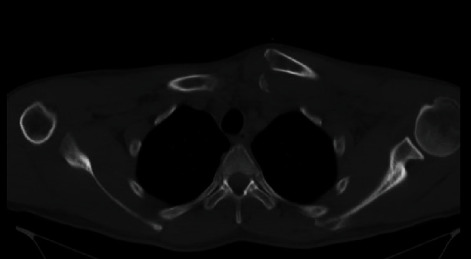
Axial CT scan demonstrating anteriorly displaced fracture dislocation of the SC joint.

**Figure 3:**
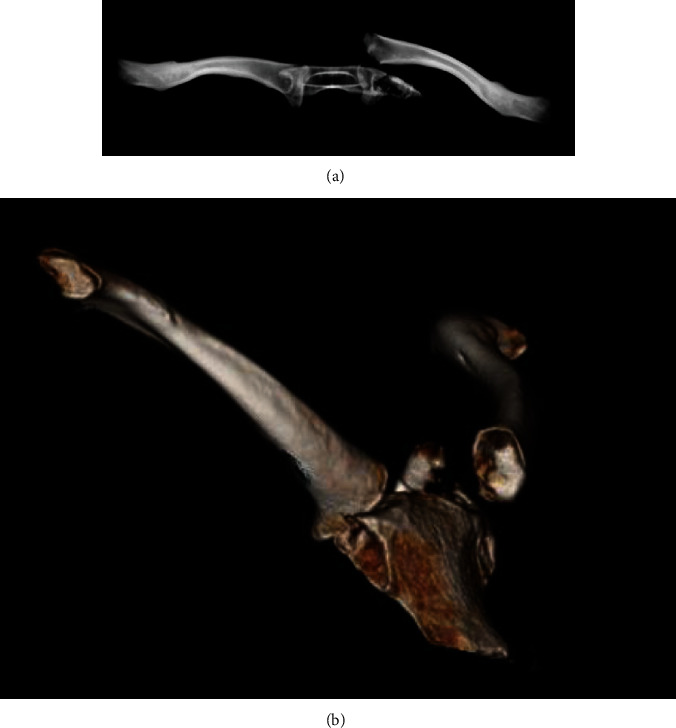
(a, b) 3D reconstructions demonstrating anterior fracture dislocation of the SC joint.

A 37-year-old right-hand-dominant male without any pertinent medical comorbidities presented to our orthopaedic department with left shoulder pain and a prominence of his sternoclavicular joint four months after being struck by a car while riding his bicycle. He was initially managed conservatively with a sling and denied any neurovascular issues. On subsequent follow-up, imaging revealed a malunion of an anteriorly displaced SCJ fracture dislocation and a Rockwood type IV acromioclavicular separation (Figures 1–3). Given the degree of pain and poor functional outcome, the patient elected to proceed with open reduction and reconstruction of his ACJ and SCJ.

### 2.1. Surgical Procedure

Intraoperatively, the patient was placed upright in the beach-chair position. The Rockwood type IV acromioclavicular separation was addressed first by starting laterally with a vertical incision over the ACJ. The posteriorly dislocated clavicle was identified and carefully mobilized from the surrounding trapezius and reduced to a reasonable position confirmed by fluoroscopy. Next, the surgical incision was then carried horizontally across the clavicle to the SCJ and the manubrium. Careful dissection revealed a fibrous union between the SCJ and the medial end of the clavicle, which was anteriorly dislocated. Following resection of the fibrous tissue, the medial edge of the clavicle was partially resected to facilitate reduction back to the residual physeal scar. For maintenance of reduction, we reconstructed the anterior sternoclavicular ligament by using a semitendinosus allograft fashioned in a figure-of-eight type weave between the manubrium and clavicle. After completion of this step, the ACJ reduction was performed by passing a TightRope (Arthex®) from the lateral clavicle through the coracoid base under fluoroscopic guidance. Once the TightRope (Arthex®) was fully engaged, our fixation was deemed stable, and the surgical incision was closed in a sequential fashion.

### 2.2. Postoperative Course

**Figure 4:**
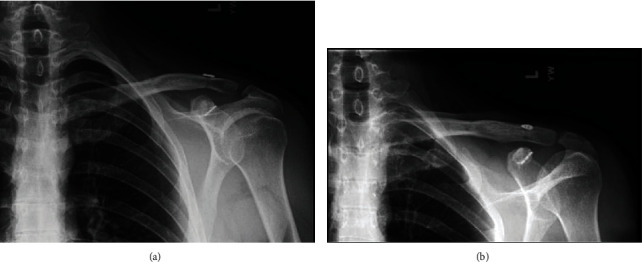
(a, b) Plain radiographs demonstrating well-aligned acromioclavicular and sternoclavicular joints without hardware complication.

The patient had no perioperative complications and was discharged from the hospital without issues. He was briefly immobilized for two weeks and then started range-of-motion training followed by strengthening. At his last six-month follow-up visit, the patient was very satisfied with his functional and clinical outcome. He reported minimal pain and only some stiffness with abduction of his shoulder. Overall, the patient maintained reduction and returned to working and recreational activities (Figures 4(a) and 4(b)).

## 3. Discussion

Bipolar clavicle dislocations, also known as traumatic “floating clavicles,” are rare with fewer than 30 documented cases [1–3, 8]. While most cases are the result of high-energy trauma, such as motor vehicle collisions, other studies have reported this injury in low-energy mechanisms such as falls from standing [1–3, 9–11]. Our case report highlights the infrequency of this injury and presents a novel surgical treatment option that has not been described in the literature.

The exact mechanism of bipolar clavicle dislocations remains uncertain; however, some authors have proposed multidirectional forces as a cause. Maruyama et al. suggested that the first rib plays a vital role in the pathophysiology of this injury, in which it acts as a pivot rotational point for the clavicle [2, 12, 13]. A strong posteromedial force on the anterolateral aspect of the clavicle could result in the tearing of acromioclavicular and coracoclavicular ligaments, dislocating the ACJ posteriorly [14]. In turn, the SCJ would torque and pivot anteriorly, resulting in disruption of the surrounding ligamentous structures between the manubrium and the clavicle, such as the costoclavicular, interclavicular, and capsular ligaments [15]. Furthermore, Maruyama et al. suggested that an inferior force on the acromion may provoke separation of the ACJ joint [12].

Understanding the forces at play is critical for management; however, there is no ideal treatment protocol for bipolar clavicle dislocations. Approaches have ranged from observation, isolated surgical treatment of the ACJ, to operative fixation of both ACJ and SCJ. In their series of 26 patients, Okano et al. reported that operative management yielded superior outcomes compared to purely conservative treatment. A significant percentage of these operative patients failed initial conservative management due to pain and decreased functional status, [2]. Sanders et al. also reported that a majority of their patients were symptomatic following conservative management [2, 16]. Even in the delayed setting, surgical treatment has shown good clinical outcomes despite timing of initial injury, preoperative functional status, or injury pattern [2].

Our patient's injury pattern was an anterior fracture dislocation of the SCJ and a Rockwood type IV ACJ separation. Studies have only reported a total of four cases with this particular type of injury pattern and discussed various surgical methods, such as surgical hook plates, that led to favorable results [1, 8, 16, 17]. We elected to treat the ACJ separation with the Arthex® TightRope technique given its favorable track record in management of isolated ACJ separation even though it has not been described for bipolar clavicle injuries [18]. Regarding treatment of the SCJ, the optimal treatment is still controversial. Eskola advocates for open treatment of SCJ dislocations due to the high redislocation rate and poor clinical outcomes from closed reduction [19]. With open treatment, there are several surgical techniques which include K-wires [3, 4], cerclage wire [5], compression screws [6], polyester mesh [7], and FiberWire [2]. In our case report, we treated the SCJ separation with a semitendinosus allograft, which has not been described for bipolar clavicle dislocations. Castropil et al. reported successful outcomes with semitendinosus allografts for treatment of isolated SCJ dislocations [20]. Tendon allografts may be a favorable alternative to metal hardware, such as K-wires since there have been several reports of cardiac tamponade and tracheoinnominate artery fistula resulting from the migration of K-wires [14, 21, 22]. Also, a tendon allograft reliably provides flexibility and stability for the treatment of these difficult injuries.

## 4. Conclusion

We present a rare case of a bipolar clavicle dislocation, and optimal treatment for these injuries remains controversial. The current study described the first successful treatment using a semitendinosus allograft to reconstruct the SCJ and an Arthex® TightRope to maintain the ACJ reduction.
